# List of Accidents, Admitted into the Pennsylvania Hospital, from April 4th to April 18th, 1838

**Published:** 1838-04-25

**Authors:** 


*List of Accidents, admitted into the Pennsylvania Hospital, from April 4th to April 18th,* 1838.

One case of badly lacerated wound of the scalp, caused by a fall from a carrriage; dressed with adhesive plaster; was attacked on the third day with erysipelas, and sloughed; sloughing since ceased, and wound is at present closing by granulation. One case of fracture of the skull, conjoined with fracture of both fore arms, caused by a fall from the rigging of a vessel—died in five days from meningitis. One case of injury from blasting. The face was badly burnt, eyes filled with sand, particles of which had penetrated the cornea. The left humerus was broken above the condyles, the olecranon fractured, and a portion of it removed previous to admisson. The joint was so much injured that amputation was performed eighteen hours after his entrance. The arm is doing well, inflammation of the eyes much reduced, and burns healing. One case of fractured clavicle—dressed with the usual apparatus, and doing well. One case of contusion of the abdomen, and strain, followed by orchitis. Leeches were applied to the testicle, and the patient kept on his back with warm cloths to the abdomen—almost cured. One case of punctured wound of the leg, just below the knee joint, caused by a fall on a nail; dressed with fracture box and poultice—purged, and doing well. One case of extensive burn, in a woman eight months gone with child—caused by her clothes taking fire. The *whole body and extremities*, with the exception of the legs, from the knee down, were burnt to a crisp. Dressed with hot poultices, and bottles of hot water to the feet—hot brandy and water every half hour. Labour came on, but the patient died three hours after admission. The Cæsarean section was performed by Dr. Randolph, within a few minutes after death; but the child was found dead. One case of lacerated finger, from an injury by a rail-road car—dressed with simple dressing and a splint.

The case of fractured fibula, reported in the last, was cured in twenty-three days—no deformity. The gun-shot wound of the elbow has been twice attacked by erysipelas, and sloughed extensively—at present under treatment.

